# Safety and immunogenicity of a meningococcal B recombinant vaccine when administered with routine vaccines to healthy infants in Taiwan: A phase 3, open-label, randomized study

**DOI:** 10.1080/21645515.2018.1425659

**Published:** 2018-02-15

**Authors:** Nan-Chang Chiu, Li-Min Huang, Arnold Willemsen, Chiranjiwi Bhusal, Ashwani Kumar Arora, Zenaida Reynoso Mojares, Daniela Toneatto

**Affiliations:** a Department of Pediatrics, MacKay Children's Hospital, Taipei, Taiwan; b Mackay Junior College of Medicine, Nursing, and Management, Taipei, Taiwan; c Mackay Medical College, Taipei, Taiwan; d Department of Pediatrics, National Taiwan University Children's Hospital, National Taiwan University College of Medicine, Taipei, Taiwan; e Plus100 B.V. c/o Biostatistics, GSK, Amsterdam, the Netherlands; f Research and Development Center, GSK, Siena, Italy

**Keywords:** *Neisseria meningitidis*, serogroup B meningococcal vaccine, immunogenicity, co-administration, safety, infant

## Abstract

*Neisseria meningitidis* is associated with high mortality and morbidity in infants and children worldwide. This phase 3 study (NCT02173704) evaluated safety and immunogenicity of a 4-component serogroup B recombinant meningococcal vaccine (4CMenB) co-administered with routine vaccines in Taiwanese infants. In total, 225 healthy infants were randomized (2 : 1 ) to receive 4CMenB and routine vaccines (4CMenB+Routine) or routine vaccines only (Routine group) at 2, 4, 6 and 12 months of age. Routine vaccines were diphtheria-tetanus-acellular pertussis-inactivated poliovirus*-Haemophilus influenzae* type b, 13-valent pneumococcal, hepatitis B, measles-mumps-rubella and varicella vaccines. Immune responses to 4CMenB components (factor H binding protein [fHbp], Neisserial adhesin A [NadA], porin A [PorA] and *Neisseria* heparin-binding antigen [NHBA]) were evaluated at 1 month post-primary and post-booster vaccination, using human serum bactericidal assay (hSBA). Reactogenicity and safety were also assessed. A sufficient immune response was demonstrated for fHbp, NadA and PorA, at 1 month post-primary and booster vaccination. In the 4CMenB+Routine group, hSBA titers ≥5 were observed in all infants for fHbp and NadA, in 79% and 59% of infants for PorA and NHBA, respectively, at 1 month post-primary vaccination and in 92–99% of infants for all antigens, at 1 month post-booster vaccination. In the 4CMenB+Routine group, hSBA geometric mean titers for all antigens increased post-primary (8.41–963) and post-booster vaccination (17–2315) compared to baseline (1.01–1.36). Immunogenicity of 4CMenB was not impacted by co-administration with routine pediatric vaccines in infants. Reactogenicity was slightly higher in the 4CMenB+Routine group compared with Routine group, but no safety concerns were identified.

## Introduction

Invasive meningococcal disease (IMD) is caused by *Neisseria meningitidis* and is associated with high morbidity and mortality in children ≤5 years of age.^1^^,^^2^ Among the 12 known serogroups of *N. meningitidis*, 6 ( A, B, C, W, X and Y) account for nearly all meningococcal disease worldwide.^3^

Following the introduction of meningococcal vaccination (monovalent or quadrivalent conjugate vaccines against serogroups A, C, W and Y) in national immunization programs worldwide, the incidence and epidemiology of IMD changed over the last decade, with declines in the prevalence of serogroups A (particularly in the African meningitis belt where mass vaccination campaigns were successfully implemented), and C and Y (in Europe and the United States).^4^ However, serogroup B persists^5^ and is now the prominent cause of IMD in Australia, New Zealand,^6^ Northern Africa,^7^ Europe^8^ and the United States^9^ and a leading cause along serogroup C in Latin America.^10^

In Taiwan, levels of IMD are low; however, an incidence of 0.013 to 0.034 per 100,000 persons was reported between 2011 and 2016, corresponding to 3–8 confirmed meningococcal cases each year. The majority of cases are attributed to serogroup B (67%), with serogroups C (11%) and Y (3%) having a lower prevalence.^11^ At present, meningococcal vaccination is not included in the immunization program in Taiwan, but the Taiwanese Advisory Committee on Immunization Practices recommends vaccination with a quadrivalent vaccine against serogroups A, C, W and Y for people at high-risk for disease and travelers to areas affected by IMD.^11^

The 4-component meningococcal serogroup B vaccine (4CMenB, *Bexsero*, GSK) is a recombinant protein-based vaccine targeting *N. meningitidis* serogroup B. The vaccine contains factor H-binding protein (fHbp), Neisserial adhesin A (NadA), *Neisseria* heparin-binding antigen (NHBA), and outer membrane vesicles (OMV) expressing porin A (PorA) protein.^12^ The 4 components are expressed by most serogroup B strains and the vaccine is predicted to provide 66–91% coverage against serogroup B strains worldwide.^13^

4CMenB is currently licensed in the US, Europe, Canada, Australia and some countries in South America.^12^ Vaccine effectiveness data has recently been reported for the first time in the United Kingdom, 10 months after the introduction of 4CMenB in the infant national immunization schedule. In vaccine-eligible infants below 12 months of age who received the first 2 doses of a reduced 2 + 1 schedule, the reported 4CMenB effectiveness was 82.9% against all meningococcal serogroup B cases.^14^

Currently, no meningococcal conjugate vaccine against group B is available in Taiwan. This study evaluated the safety and immunogenicity of 4CMenB when co-administered with routine vaccines to healthy Taiwanese infants at 2, 4 and 6 months of age, followed by a booster dose at 12 months of age and was designed to support licensure of 4CMenB in Taiwan.

**Figure 1.**
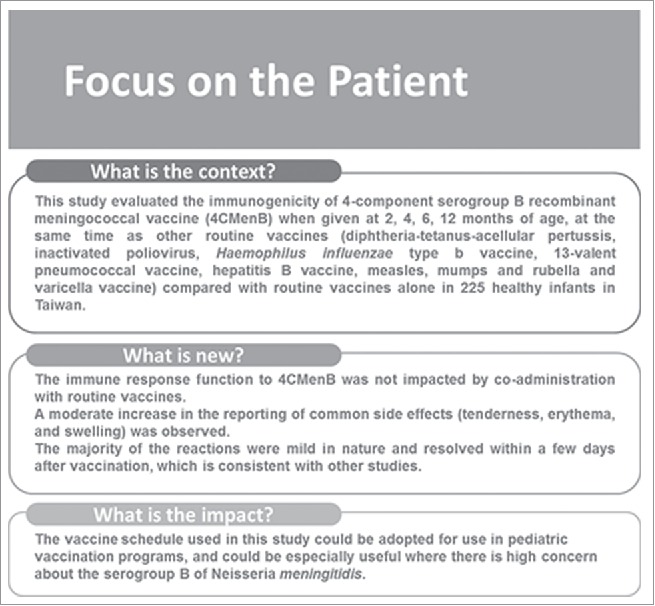
Focus on patient section

Figure 1 represents a Focus on Patient Section, which elaborates on the research clinical relevance and impact to be shared to patients by Health Care Professionals.

## Results

### Demographics

**Figure 2.**
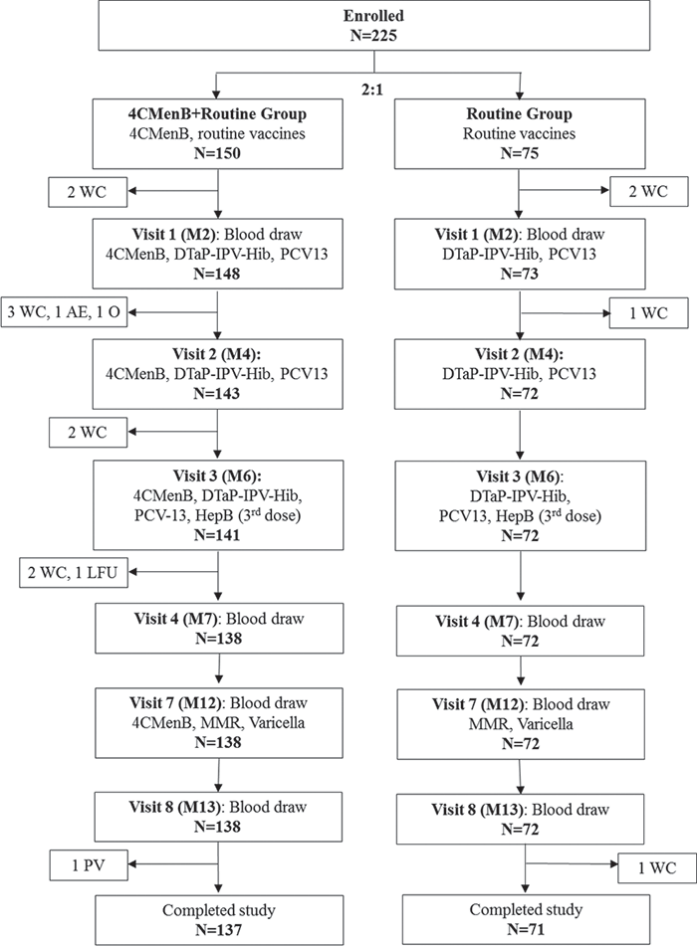
Participant flowchart. Footnotes: N, number of infants; 4CMenB, 4-component serogroup B recombinant meningococcal recombinant vaccine; M, month; DTaP-IPV-Hib, combined diphtheria, tetanus, acellular pertussis, inactivated poliovirus types 1, 2, 3 and *Haemophilus influenzae* type b vaccine, PCV13, 13-valent pneumococcal conjugate vaccine; HepB, hepatitis B vaccine; MMR, measles, mumps and rubella vaccine; Varicella, varicella vaccine; WC, withdrawal of consent; AE, adverse event; O, other; LFU, lost to follow-up; PV, protocol violation. According to the Taiwanese Immunization Program for Infants, the first 2 doses of HepB are given at 0 (M0) and 1 month (M1) of age.

A total of 225 infants were enrolled: 150 in the 4CMenB+Routine group, who received 4CMenB at 2, 4, 6 months of age and a booster dose at 12 months together with routine vaccines and 75 in the Routine group, who received routine vaccines alone. Of these, 221 were included in the full analysis set (Fig. 2). Both groups received diphtheria-tetanus-acellular pertussis, inactivated poliovirus, *Haemophilus influenzae* type b (DTaP-IPV-Hib; *Infanrix*/IPV+Hib, GSK) and 13-valent pneumococcal (PCV13; *Prevenar* 13, Pfizer) vaccines at 2, 4, 6 months of age, hepatitis B vaccine (HepB; *Engerix-B*, GSK) at 6 months, and measles, mumps, rubella (MMR; *Priorix*, GSK) and varicella (*Varilrix*, GSK) vaccines at 12 months of age.

Demographic characteristics were well-balanced across the 2 study groups. The mean age of the infants at enrollment was 68.3 days; ≥99% of infants were Asian, and 48% were girls (Supplementary Table S1).

### Immunogenicity

**Figure 3.**
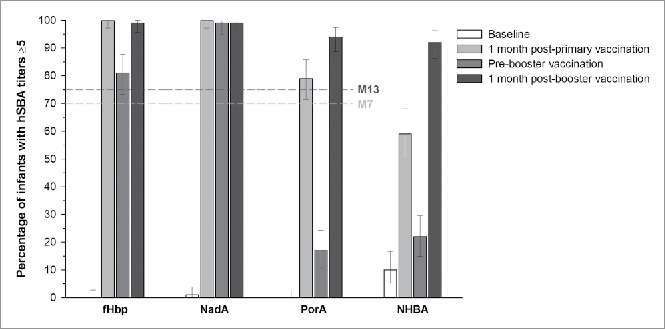
Participants in the 4CMenB+Routine group with hSBA titers ≥5 against each of the indicator strains for the four vaccine antigens (full analysis set). Footnotes: 4CMenB, 4-component serogroup B recombinant meningococcal vaccine; hSBA, human serum bactericidal assay; M, month; fHbp, factor H-binding protein; NadA, Neisserial adhesin A; PorA, porin A; NHBA, *Neisseria* heparin-binding antigen. Note: Error bars represent 95% confidence intervals. Dotted horizontal lines represent the criteria for assessment of sufficient immune response at 1 month post-primary (M7) and post-booster (M13) vaccination. The immune response was considered sufficient if the lower limit of the 95% confidence intervals for the percentage of infants with hSBA titers ≥5 post-primary or booster vaccination was above 70% at M7 or 75% at M13, respectively.

Post-primary vaccination, in the 4CMenB+Routine group, the lower limits (LLs) of the 95% confidence intervals (CIs) were 97.2%, 97.2% and 71.4% for the percentage of infants with human serum bactericidal assay (hSBA) titers ≥5 against 3 indicator strains specific for fHbp, NadA and PorA, respectively, above the pre-specified margin of 70%. Post-booster vaccination, the LLs of the 95% CIs were 95.7%, 94.7% and 88.7%, respectively, above the margin of 75%. Thus, a sufficient immune response to 4CMenB vaccination when administered with routine pediatric vaccines was demonstrated both post-primary and post-booster vaccination **(**Fig. 3).

One month post-primary vaccination, in the 4CMenB+Routine group, hSBA titers ≥5 were achieved in 100%, 100%, 79% and 59% of infants against the indicator strains for fHbp, NadA, PorA and NHBA, respectively (Fig. 3). In the Routine group, ≤8% of infants had hSBA titers ≥5 against any of the indicator strains.

Pre-booster vaccination, 81%, 99%, 17% and 22% of infants in the 4CMenB+Routine group had hSBA titers ≥5 for fHbp, NadA, PorA and NHBA, respectively. One month post-booster vaccination, hSBA titers ≥5 were observed in 99% of infants for fHbp and NadA, 94% of infants for PorA and in 92% of infants for NHBA (Fig. 3). In the Routine group, ≤13% of infants had hSBA titers ≥5 against all indicator strains post-booster vaccination.

The percentages of infants with hSBA antibody titers ≥4 for all vaccine components are presented in Supplementary Figure S1.

In both groups, pre-vaccination hSBA geometric mean titers (GMTs) against all serogroup B indicator strains were ≤1.75 in both groups. One month post-primary vaccination, in the 4CMenB+Routine group, antibody GMTs against indicator strains increased 71-, 875-, 9.18- and 5.69-fold for fHbp, NadA, PorA and NHBA, respectively. hSBA GMTs for all vaccine components decreased at the pre-booster timepoint, but remained above baseline levels, and increased 155-, 2110-, 25-, and 12-fold compared to baseline, for fHbp, NadA, PorA and NHBA, respectively, following booster vaccination (Table 1). In the Routine group, no increase from baseline levels was observed in hSBA GMTs against serogroup B indicator strains, throughout the study (Table 1).

**Table 1.** hSBA GMTs for 4CMenB components (full analysis set)

	4CMenB+Routine Group		Routine Group	
	n	GMT/*GMR* (95% CI)	n	GMT/*GMR* (95% CI)
fHbp				
Baseline (pre-vaccination)	132	1.01 (0.99–1.02)	65	1.02 (0.98–1.05)
1 month post-primary vaccination	130	72 (64–81)	66	1.01 (0.99–1.03)
*GMR (1 month post-primary/baseline)*	*116*	*71 (63–80)*	*59*	*0.99 (0.95–1.04)*
Pre-booster vaccination	123	11 (9.27–13)	64	1.26 (1.06–1.50)
1 month post-booster vaccination	127	157 (131–188)	64	1.1 (1.01–1.19)
*GMR (1 month post-booster/baseline)*	*113*	*155 (127–188)*	*56*	*1.07 (0.97–1.19)*
NadA				
Baseline (pre-vaccination)	141	1.08 (0.98–1.18)	72	1.03 (0.98–1.08)
1 month post-primary vaccination	129	963 (864–1073)	70	1.00 (1.00–1.00)
*GMR (1 month post-primary/baseline)*	*124*	*875 (752–1019)*	*69*	*0.97 (0.93–1.03)*
Pre-booster vaccination	135	205 (174–242)	70	1.10 (0.91–1.33)
1 month post-booster vaccination	134	2315 (1893–2832)	71	1.00 (1.00–1.00)
*GMR (1 month post-booster/baseline)*	*129*	*2110 (1674–2659)*	*70*	*0.97 (0.93–1.03)*
PorA				
Baseline (pre-vaccination)	145	1.01(0.99–1.02)	73	1.00 (1.00–1.00)
1 month post-primary vaccination	135	9.20 (7.82–11.00)	71	1.02 (0.98–1.05)
*GMR (1 month post-primary/baseline)*	*134*	*9.18(7.79–11.00)*	*71*	*1.02 (0.98–1.05)*
Pre-booster vaccination	136	1.91 (1.63–2.25)	70	1.04 (0.98–1.10)
1 month post-booster vaccination	136	26 (21–31)	71	1.00 (1.00–1.00)
*GMR (1 month post-booster/baseline)*	*135*	*25 (21–31)*	*71*	*1.00 (1.00–1.00)*
NHBA				
Baseline (pre-vaccination)	121	1.36 (1.20–1.54)	64	1.75 (1.37–2.24)
1 month post-primary vaccination	*123*	*8.41 (6.63–11.00)*	*65*	*1.25 (1.08–1.46)*
*GMR (1 month post-primary/baseline)*	100	5.69 (4.25–7.61)	57	0.68 (0.54–0.85)
Pre-booster vaccination	130	2.18 (1.81–2.63)	65	1.60 (1.34–1.91)
1 month post-booster vaccination	130	17 (14–20)	69	1.58 (1.33–1.89)
*GMR (1 month post-booster/baseline)*	*105*	*12 (8.99–15.00)*	*60*	*0.91 (0.68–1.21)*

hSBA, human serum bactericidal assay; GMT, geometric mean titer; 4CMenB, 4-component serogroup B recombinant meningococcal vaccine; n, number of infants with available results; GMR, geometric mean ratio; CI, confidence interval; fHbp, factor H-binding protein; NadA, Neisserial adhesin A; PorA, porin A; NHBA, *Neisseria* heparin-binding antigen.

### Reactogenicity and safety

In general, all infants in the 4CMenB+Routine group and 93% of infants in the Routine group experienced at least 1 solicited AE, regardless of the vaccination. The percentage of infants experiencing at least 1 solicited AE tended to decrease from the first to subsequent vaccinations, both in the 4CMenB+Routine group (95%, 91%, 90% and 88% for first, second, third and booster vaccination, respectively) and the Routine group (79%, 76%, 74% and 56% for first, second, third and booster vaccination, respectively).

In the 4CMenB+Routine group, the most frequently reported solicited local AEs were injection site tenderness (for 48%–51% of infants) after each 4CMenB vaccination and injection site tenderness (for 27%–34% of infants) after each of the routine vaccinations. Injection site tenderness was the only severe solicited local AE reported in the 4CMenB+Routine group, after each vaccination with 4CMenB (≤5%) or routine vaccines (≤2%) (Table 2). In the Routine group, the most frequently reported solicited local AEs were injection site induration (8%–24% of infants) and erythema (4%–21% of infants) (Table 2).

**Table 2.** Number and percentage of infants with local adverse events after each dose and each vaccine, reported in the 7-day period post-vaccination (solicited safety set).

	Tenderness		Severe tenderness	Erythema		Swelling		Induration	
	4CMenB +Routine	Routine	4CMenB +Routine	4CMenB +Routine	Routine	4CMenB +Routine	Routine	4CMenB +Routine	Routine
1^st^ dose	N=148	N=73	N=148	N=148	N=73	N=148	N=73	N=148	N=73
4CMenB	75 (51%)	—	8 (5%)	54 (36%)	—	34 (23%)	—	63 (43%)	
PCV13	50 (34%)	12 (16%)	3 (2%)	26 (18%)	9 (12%)	11 (7%)	4 (5%)	29 (20%)	15 (21%)
DTaP-IPV-Hib	45 (31%)	12 (16%)	2 (1%)	26 (18%)	3 (4%)	15 (10%)	3 (4%)	23 (16%)	11 (15%)
2^nd^ dose	N=140	N=72	N=140	N=140	N=72	N=140	N=72	N=140	N=72
4CMenB	67 (48%)	—	5 (4%)	59 (42%)	—	35 (25%)	—	51 (36%)	
PCV13	44 (31%)	9 (13%)	3 (2%)	35 (25%)	12 (17%)	12 (9%)	6 (8%)	18 (13%)	8 (11%)
DTaP-IPV-Hib	45 (32%)	10 (14%)	3 (2%)	39 (28%)	12 (17%)	18 (13%)	6 (8%)	23 (16%)	16 (22%)
3^rd^ dose	N=138	N=72	N=138	N=138	N=72	N=138	N=72	N=138	N=72
4CMenB	68 (49%)	—	6 (4%)	55 (40%)	—	49 (36%)	—	57 (41%)	—
PCV13	45 (33%)	9 (13%)	3 (2%)	27 (20%)	12 (17%)	17 (12%)	4 (6%)	21 (15%)	11 (15%)
DTaP-IPV-Hib	42 (30%)	8 (11%)	2 (1%)	28 (20%)	15 (21%)	20 (14%)	10 (14%)	25 (18%)	16 (22%)
HepB	45 (33%)	7 (10%)	3 (2%)	33 (24%)	12 (17%)	25 (18%)	6 (8%)	33 (24%)	17 (24%)
Booster dose	N=137	N=72	N=137	N=137	N=72	N=137	N=72	N=137	N=72
4CMenB	66 (49%)^*^		6 (4%)	53 (39%)		44 (33%)		56 (41%)	
MMR	37 (27%)	6 (8%)	3 (2%)	24 (18%)	13 (18%)	19 (14%)	4 (6%)	15 (11%)	6 (8%)
Varicella	41 (30%)	5 (7%)	3 (2%)	32 (23%)	14 (19%)	21 (15%)	6 (8%)	14 (10%)	7 (10%)

4CMenB, 4-component serogroup B recombinant meningococcal vaccine; N, number of exposed infants; DTaP-IPV-Hib, combined diphtheria, tetanus, acellular pertussis, inactivated poliovirus types 1, 2, 3 and *Haemophilus influenzae* type b vaccine; PCV13, 13-valent pneumococcal conjugate vaccine; HepB, hepatitis B vaccine; MMR, measles, mumps and rubella vaccine; Varicella, varicella vaccine.

Note:

* N=136

The most frequently reported solicited systemic AE after each vaccination was irritability in both the 4CMenB+Routine and Routine groups, reported for 52%–75% and 22–44%) of infants, respectively (Table 3). After each vaccination in the 4CMenB+Routine group, fever (temperature ≥38°C) was reported by a higher percentage of infants (44%–51%) than in the Routine group (8%–17%) (Table 3). Severe fever (temperature ≥40°C) was reported for one infant in the 4CMenB+Routine group after second vaccination and for one in each group after booster vaccination. Medically-attended fever was reported for 1 (1%), 3 (2%) and 4 (3%) infants after the first, third and the booster dose in the 4CMenB+Routine group and for 2 (3%) infants after the booster dose in the Routine group. Most solicited systemic AEs were mild to moderate in intensity.

**Table 3.** Number and percentage of infants with systemic reactions and fever after each dose reported in the 7-day period post-vaccination (solicited safety set).

	4CMenB+Routine				Routine			
	1st dose	2nd dose	3rd dose	Booster dose	1st dose	2nd dose	3rd dose	Booster dose
Changing in eating habits	92 (62%)	72 (51%)	72 (52%)	58 (42%)	26 (36%)	21 (29%)	17 (24%)	19 (26%)
Sleepiness	79 (53%)	66 (47%)	49 (36%)	36 (26%)	33 (45%)	21 (29%)	13 (18%)	9 (13%)
Irritability	111 (75%)	90 (64%)	94 (68%)	71 (52%)	32 (44%)	31 (43%)	28 (39%)	16 (22%)
Persistent crying	96 (65%)	82 (59%)	72 (52%)	58 (42%)	30 (41%)	23 (32%)	28 (39%)	15 (21%)
Vomiting	21 (14%)	16 (11%)	15 (11%)	10 (7%)	6 (8%)	9 (13%)	7 (10%)	6 (8%)
Diarrhea	28 (19%)	30 (21%)	19 (14%)	27 (20%)	8 (11%)	9 (13%)	4 (6%)	10 (14%)
Rash	18 (12%)	18 (13%)	16 (12%)	25 (18%)	4 (5%)	8 (11%)	4 (6%)	9 (13%)
Fever^*^ ≥38.0 °C	71 (48%)	72 (51%)	68 (49%)	60 (44%)	11 (15%)	11 (15%)	12 (17%)	6 (8%)
Fever ≥40.0 °C	0	1 (1%)	0	1 (1%)	0	0	0	1 (1%)
Medically-attended fever	1 (1%)	0	3 (2%)	4 (3%)	1 (1%)	0	0	2 (3%)
Analgesic/antipyretic treatment used	35 (24%)^**^	38 (27%)	30 (22%)	20 (15%)	1 (1%)	2 (3%)^**^	5 (7%)	3 (4%)

4CMenB, 4-component serogroup B recombinant meningococcal vaccine.

Note:

* Body temperature (°C) was solicited but it was not reported as a systemic adverse event in this study.

** Data reported for N-1 children (N, number of exposed infants).

The most frequently reported symptoms during a 28-day period post-MMR/varicella vaccination were rash for 36% and 33% of infants in the 4CMenB+Routine and Routine groups, respectively. Lymphadenopathy (parotid/salivary gland swelling) was reported for 25% of infants in group 4CMeB+ Routine and 29% of infants in the Routine group). Rash was the most reported severe symptom in 4% of infants in the 4CMenB+Routine group and 7% of infants in Routine group. Fever and severe fever was reported in 66% and 1% of infants in the 4CMenB+Routine respectively, and in 46% and 7% of infants in the Routine group, respectively. Overall, 74% and 62% of infants in the 4CMenB+Routine and Routine groups, respectively, received paracetamol therapeutically.

Overall, following any vaccination, any unsolicited AE was reported for 72% and 42% of infants in groups 4CMenB+Routine and Routine, respectively. Any probably or possibly related AEs were reported for 59% and 24% of infants in groups 4CMenB+Routine and Routine and the majority were due to solicited AEs ongoing after the 7-day reporting period. The incidence of medically-attended AEs was similar between groups (Supplementary Table S2).

One infant from 4CMenB+Routine group was withdrawn from the study on day 57 due to an AE (congenital cystic kidney disease and renal dysplasia) which was not considered related to the study vaccines.

At least one serious AE (SAE) was reported for 21 infants: 13 in the 4CMenB+Routine group (9%) and 8 in the Routine group (11%) and all were resolved before study end. No SAEs were considered as possibly or probably related to the study vaccine.

## Discussion

The study demonstrated a robust immune response to 4CMenB vaccine, when given concomitantly with routine vaccines to healthy infants at 2, 4, 6 and 12 months of age, in terms of percentage of infants achieving hSBA titers ≥5 against indicator strains for fHbp, NadA and PorA at 1 month post-primary and post-booster vaccination.

Our results are in line with previous studies, which demonstrated a sufficient immune response following primary and booster vaccination of infants with 4CMenB, when co-administered with routine pediatric vaccines and according to various schedules.^15-18^ The same criteria to assess sufficient immune response to 4CMenB were used in our study and endorsed by the European Medicines Agency during the 4CMenB clinical development phase.^15^ However, no suitable indicator strain had been identified for NHBA at the time, and therefore the assessment of a sufficient immune response was only planned for fHbp, NadA and PorA.

The majority of infants in the 4CMenB+Routine group achieved hSBA titers ≥5 against all 4 serogroup B indicator strains after both primary and booster vaccination. At 1 month after primary vaccination, hSBA GMTs for all vaccine components increased significantly from pre-vaccination levels, with fold-increases similar to those observed in European infants following the administration of a 3-dose primary series of 4CMenB with routine vaccines.^18^ As expected, GMTs waned by 12 months of age, but remained above baseline levels and were significantly increased following the booster dose. Our results are consistent with those from previous studies where 4CMenB was administered with routine vaccines and similar schedules where most of infants achieved hSBA titers ≥5 against indicator strains for fHbp NadA and PorA, post-primary vaccination^16^^,^^18^ and for fHbp and NadA, post-booster dose.^18^

The observed GMT increase from baseline levels was higher following the booster dose than following primary vaccination, suggesting a boosting of the immune response. This confirms previous results reported in European infants, for whom booster responses were observed after the administration of 4CMenB according to similar schedules.^18^^,^^19^ A recent persistence study, following 4CMenB booster vaccinations at 12, 18 or 24 months reported that antibody concentration waned after 12–36 months regardless of the timing of boosters, but an additional 4CMenB dose given at 4 years of age induced a robust memory response.^20^

The tolerability profile of 4CMenB vaccine was acceptable, with no major safety concerns that were identified when administered concomitantly with routine vaccinations. The majority of the reactions was mild in nature and resolved within a few days after vaccination. These results were consistent with what was observed in studies with infants of similar age.^17^^,^^18^

The incidence of AEs following co-administration of 4CMenB and routine vaccines was comparable with previous reports for infants.^16^^,^^18^ The percentage of infants in the 4CMenB+Routine group with fever ≥38°C after any dose (44%–51%) seemed lower than in another study where infants were vaccinated with 4CMenB, DTaP-HBV-IPV/Hib and 7-valent PCV (57%–77%), and similar to the percentage of infants reporting fever when prophylactic paracetamol was administered with 4CMenB, DTaP-HepB-IPV/Hib and 7-valent PCV (37%–60%).^21^

This is the first study to report on 4CMenB administration to Asian infants, with the only other 4CMenB vaccination trial being carried out among Korean adolescents.^22^ Therefore, our results provide the basis for comparisons with other age groups and geographical regions and contribute to the generalization of 4CMenB vaccination data to the Asian population. The incidence of IMD peaks in infancy,^4^ with case fatality ratios of up to 8.1% being attributed to serogroup B-caused IMD in children <1 year of age.^7^ In view of the effectiveness data emerging following the introduction of 4CMenB in the national immunization program in the United Kingdom,^14^ the implementation of a similar measure in Taiwan could have an important impact. Our study shows that 4CMenB can be safely administered to Taiwanese infants together with routine immunizations, which would allow the administration of the vaccine without increasing the number of visits and associated costs.

The trial was designed with sufficient power to assess the confirmatory objectives and to comply with the current pediatric vaccination scheme in Taiwan. However, there are potential limitations. A relatively low number of infants were evaluated, compared with other similar meningococcal studies where up to 3,630 infants were enrolled.^18^ As is the case for all meningococcal vaccine trials, efficacy cannot be measured due to the low incidence of IMD and the low sample size further limits the evaluation of 4CMenB vaccination against IMD caused by serogroup B in Taiwan. Although hSBA titers ≥4 against serogroup B tested strains are usually considered protective, a serological correlate of protection was only clearly established for serogroup C.^23^ Another potential limitation is the fact that immune responses to the routine vaccines were not measured in our study. However, a similar study showed that immunogenicity of DTaP-HepB-IPV/Hib was not impacted by co-administration with 4CMenB.^18^ Additionally, the open-label design may have impacted the safety data towards higher AE reporting rates.

## Conclusions

The 4CMenB vaccine showed a robust immune response against strains specific for each of the vaccine components (fHbp, NadA, PorA and NHBA) and an acceptable safety and tolerability profile similar to that seen in other clinical trials. The vaccination schedule used in the study, which was in line with the current schedule of the Taiwan National Immunization Program, has the potential to be adopted as an add-on to the currently recommended pediatric vaccination schedule once the 4CMenB vaccine is licensed in Taiwan.

## Methods

### Study design and participants

This phase 3, randomized, controlled, open-label study with 2 parallel groups was conducted in healthy infants between September 2014 and June 2016, at 2 centers in Taiwan.

Infants were healthy 2 month-olds (55–89 days) at the time of enrollment, born after a gestation period of ≥37 weeks and with a birth weight of ≥2.5 kg, and were available for all the scheduled study visits. Criteria of exclusion from study enrollment are presented in Supplementary Text S1.

The infants were randomized in a 2:1 ratio to receive either 4CMenB with routine vaccines (4CMenB+Routine group) or routine vaccines alone (Routine group). 4CMenB was administered as a 3-dose primary vaccination at 2, 4 and 6 months of age followed by a booster dose at 12 months of age. Routine vaccines were (co-) administered according to the National Immunization Program in Taiwan as follows: DTaP-IPV-Hib and PCV13 at 2, 4 and 6 months of age, HepB vaccine at 6 months of age and MMR and varicella vaccine at 12 months of age. After the study, at 15 months of age, all participants were offered a booster dose of PCV13 to complete the recommended vaccination schedule.

Randomization was done accounting for center, using a web-based block randomization system.

Written informed consent was obtained from each parent/legally acceptable representative before vaccination. The study was conducted in accordance with Good Clinical Practice and the Declaration of Helsinki, and is registered at www.Clinicaltrials.gov (NCT02173704). A summary of the protocol is available at http://www.gsk-clinicalstudyregister.com (study ID 205249).

### Study vaccines

Each dose of 4CMenB contains 50 µg of the following *N. meningitidis* purified antigens: 961c (NadA), 936–741 (fHbp) and 287–953 (NHBA); 25 µg of OMV from *N. meningitidis* strain NZ98/254 (PorA), and aluminium hydroxide; sodium chloride, sucrose; histidine and water up to 5 ml.

The routine vaccines used in this study were commercial preparations, and were prepared/used according to instructions.

All vaccines were administered in the anterolateral area of the right or left thigh, intramuscularly (4CMenB, DTaP-IPV-Hib, PCV13 and HepB) or subcutaneously (MMR and varicella).

### Study objectives

The primary objective was to demonstrate sufficient immune response to 4CMenB co-administered with routine vaccines at 1 month post-primary vaccination, as measured by the percentage of infants with hSBA titer ≥1:5 against indicator strains for fHbp, NadA and PorA.

Secondary objectives were to demonstrate sufficient immune response to 4CMenB co-administered with routine vaccines evaluated at 1 month post-booster, measured by percentage of infants with hSBA titer ≥1:5 against indicator strains for fHbp, NadA and PorA (confirmatory objective) and to assess bactericidal antibodies against indicator strains for fHbp, NadA, PorA and NHBA at baseline (pre-vaccination, 2 months of age), 1 month post-primary vaccination (7 months of age), prior to the booster dose (12 months of age) and 1 month post-booster dose (13 months of age). The reactogenicity and safety of 4CMenB co-administered with routine vaccines and of routine vaccines were also evaluated.

### Immunogenicity assessments

Blood samples (5 ml) were collected pre-vaccination (M2), 1 month post-primary vaccination (M7), pre-booster dose (M12) and 1 month post-booster vaccination (M13) (Fig. 2).

Immune responses to 4CMenB were assessed using hSBA against 4 meningococcal B indicator strains: H44/76 (fHbp), 5/99 (NadA), NZ98/254 (PorA) and M10713 (NHBA), conducted at the GSK Biologicals Clinical Laboratory Science, Marburg, Germany. The percentages of infants with hSBA titers ≥4 and ≥5, and hSBA GMTs were assessed at each timepoint. An hSBA titers ≥4 is the accepted surrogate of protection against serogroup C-caused meningococcal disease and this threshold is routinely extended to the other serogroups.^9^ However, a more conservative threshold of ≥5 ensures with 95% confidence that the titer achieved is ≥4 and therefore, both endpoints were evaluated here. Geometric mean ratios (GMRs) relative to pre-vaccination levels were also calculated at M7 and M13.

### Safety assessment

All infants were observed for 30 minutes after each vaccination for any immediate reactions. Solicited local AEs (injection site erythema, induration, tenderness and swelling), solicited systemic or general AEs (body temperature ≥38°C, change in eating habits, sleepiness, irritability, persistent crying, vomiting, diarrhea and rash) and unsolicited AEs were recorded using diary cards by the childrens' parents or legally accepted representatives for 7 days after each vaccination. Fever, rash, parotid/salivary gland swelling were collected for an extended period of 28 days after the administration of MMR and varicella vaccines.

All solicited local and systemic AEs were summarized according to defined severity grading scales.

SAEs, medically attended AEs and AEs leading to withdrawal from the study were recorded throughout the entire study.

### Statistical analysis

For the 4CMenB+Routine group, a sample size of 120 evaluable infants was calculated, assuming that the proportion of infants achieving hSBA titers ≥5 would be 84%, 99% and 99% for the PorA, fHbp, and NadA strains, respectively, at 1 month after primary vaccination, based on results from a previous trial.^18^ The power to reject the null hypothesis associated with the primary objective and demonstrate sufficient response was 94%, 99% and 99%, respectively (calculated using an exact test for a single binomial proportion and a 2-sided alpha error of 0.05). Assuming the results for the 3 strains to be independent, the overall power to demonstrate sufficiency of response was 92%.

The primary objective was considered met if the LL of the 2-sided 95% CI for the percentage of infants with hSBA titer ≥5 against the indicator strains for fHbp, NadA and PorA post-primary vaccination was >70%. The secondary confirmatory objective was considered met if the LL of the 2-sided 95% CI for the percentage of infants with hSBA titer ≥5 against the indicator strains for fHbp, NadA and PorA post-booster was >75%.

Safety and immunogenicity analyses were carried out on the full analysis set at each timepoint, which included exposed participants with evaluable safety and immunogenicity data, respectively. Additional analyses to evaluate immune response to 4CMenB vaccination were performed on the per-protocol set (Supplementary Table S3).

Titers below the limit of quantification (1:2) were set to half that limit, for the quantitative analysis purpose. Unadjusted GMTs and GMRs were constructed by exponentiating the means of the log_10_-transformed hSBA titers with corresponding 2-sided 95% CIs (computed using the Clopper-Pearson method^24^).

## Trademark statement

*Bexsero, Infanrix, Engerix-B, Priorix* and *Varilrix* are trademarks owned by the GSK group of companies. *Prevenar 13* is a trademark of Pfizer.

## Supplementary Material

KHVI_A_1425659_supplemental.docx

## Abbreviations

4CMenB: 4-component serogroup B recombinant meningococcal vaccine
AE: adverse event
CI: confidence interval
DTaP-IPV-Hib: diphtheria-tetanus-acellular pertussis, inactivated poliovirus, *Haemophilus influenzae* type b vaccine
fHbp: factor H-binding protein
GMR: geometric mean ratio
GMT: geometric mean titer
HepB: hepatitis B vaccine
hSBA: human serum bactericidal assay
IMD: invasive meningococcal disease
LL: lower limit
MMR: measles, mumps and rubella
NadA: Neisserial adhesin A
NHBA: *Neisseria* heparin-binding antigen
OMV: outer membrane vesicles
PCV13: 13-valent pneumococcal vaccine
PorA: porin A
SAE: serious adverse event
SD: standard deviation
UL: upper limit
US: United States
