# Supplementary material for: Safety and immunogenicity of a meningococcal B recombinant vaccine when administered with routine vaccines to healthy infants in Taiwan: A phase 3, open-label, randomized study
Source: Hum Vaccin Immunother. 2018 Feb 15;14(5):1075–83. doi: 10.1080/21645515.2018.1425659 (PMC5989895; doi:10.1080/21645515.2018.1425659)
Supplement: KHVI_A_1425659_supplemental.docx [file khvi-14-05-1425659-s001.docx]

# Supplementary Material

# Table S1. Demographic and baseline characteristics (enrolled set)

|  | 4CMenB+Routine | Routine | Total |
| --- | --- | --- | --- |
|  | N=150 | N=75 | N=225 |
| Age, days (mean ±SD) | 67.9 ± 6.5 | 68.9 ± 6.9 | 68.3 ± 6.60 |
| Weight, kg (mean ±SD) | 5.5 ± 0.6 | 5.7 ± 0.8 | 5.6 ± 0.68 |
| Height, cm (mean ±SD)* | 57.9 ± 2.1 | 58.6 ± 2.6 | 58.1 ± 2.30 |
| BMI, kg/m^2^ (mean ±SD)* | 16.4 ± 1.4 | 16.5 ± 1.7 | 16.5 ± 1.50 |
| Girls | 82 (55%) | 27 (36%) | 109 (48%) |
| Race, n (%) |  |  |  |
| Asian | 150 (100%) | 74 (99%) | 224 (>99%) |
| Other | - | 1 (1%) | 1 (<1%) |

4CMenB, 4 component serogroup B recombinant meningococcal vaccine; N, number of infants; SD, standard deviation; BMI, basal metabolic index; n (%), number (percentage) of infants in a given category.

Note: * the number of infants evaluated was 149 for the 4CMenB+Routine group and 74 for the Routine group.

# Table S2. Unsolicited adverse events from day 1 to day 7 (unsolicited safety set)

|  | 4CMenB+Routine | Routine | |
| --- | --- | --- | --- |
|  | n (%) | | n (%) |
| Any, post-dose 1 | 46 (32%) | 8 (11%) | |
| At least possibly related | 35 (24%) | 6 (8%) | |
| Any, post-dose 2 | 30 (21%) | 7 (10%) | |
| At least possibly related | 22 (16%) | 5 (7%) | |
| Any, post-dose 3 | 51 (37%) | 18 (25%) | |
| At least possibly related | 45 (33%) | 7 (10%) | |
| Any, post-booster | 43 (31%) | 9 (13%) | |
| At least possibly related | 37 (27%) | 4 (6%) | |
| Any, overall | 104 (72%) | 30 (42%) | |
| At least possibly related | 86 (59%) | 17 (24%) | |
| Medically attended | 131 (90%) | 66 (92%) | |
| Unsolicited AEs by System Organ Class | | | |
| General disorders and administration site conditions | 84 (58%) | 15 (21%) | |
| Infections and infestations | 20 (14%) | 13 (18%) | |
| Psychiatric disorders | 20 (14%) | 8 (11%) | |
| Skin and subcutaneous tissue disorders | 14 (10%) | 2 (3%) | |
| Gastrointestinal disorders | 9 (6%) | 2 (3%) | |
| Most frequently reported unsolicited AEs by preferred term | | | |
| Injection site induration | 75 (52%) | 12 (17%) | |
| Injection site swelling | 41 (28%) | 3 (4%) | |
| Injection site erythema | 23 (16%) | 1 (1%) | |
| Eating disorder | 17 (12%) | 6 (8%) | |
| Nasopharyngitis | 11 (8%) | 6 (8%) | |

4CMenB, 4 component serogroup B recombinant meningococcal vaccine; n (%), number (percentage) of infants for whom the AE was reported; AE, adverse event.

# Table S3. Summary of immune responses to 4CMenB vaccination (per-protocol set)

|  | 4CMenB+Routine group | | | | | Routine Group | | |  |
| --- | --- | --- | --- | --- | --- | --- | --- | --- | --- |
|  | N | % of infants with hSBA ≥5 (95% CI) | GMT (95% CI) | |  | N | % of infants with hSBA ≥5 (95% CI) | GMT (95% CI) | |
| fHbp |  | | |  | | | | |  |
| Baseline (pre-vaccination) | 103 | 0 (0–3.5) | 1.0 (1.0–1.0) | |  | 50 | 0 (0–7.1) | 1.02 (0.98–1.07) | |
| 1 month post-primary vaccination | 109 | 100 (96.7–100) | 71 (63.0–80.0) | |  | 55 | 0 (0–6.5) | 1.01 (0.99–1.04) | |
| Pre-booster vaccination | 104 | 82 (72.6–88.6) | 11 (8.95–13.0) | |  | 51 | 2 (0.05–10.4) | 1.24 (1.01–1.52) | |
| 1 month post-booster vaccination | 107 | 99 (94.9–99.98) | 155 (126.0–189.0) | |  | 54 | 2 (0.05–9.9) | 1.10 (1.00–1.20) | |
| NadA |  | | |  | | | | |  |
| Baseline (pre-vaccination) | 111 | 1 (0.02–4.9) | 1.09 (0.97–1.23) | |  | 57 | 2 (0.04–9.4) | 1.03 (0.97–1.10) | |
| 1 month post-primary vaccination | 108 | 100 (96.6–100) | 967 (858–1091) | |  | 58 | 0 (0–6.2) | 1.0 (1.0–1.0) | |
| Pre-booster vaccination | 113 | 98 (93.8–99.78) | 209 (173–253) | |  | 57 | 2 (0.04–9.4) | 1.12 (0.89–1.42) | |
| 1 month post-booster vaccination | 112 | 98 (93.7–99.78) | 2346 (1859–2961) | |  | 58 | 0 (0–6.2) | 1.0 (1.0–1.0) | |
| PorA |  | | |  | | | | |  |
| Baseline (pre-vaccination) | 115 | 0 (0–3.2) | 1.01 (0.99–1.03) | |  | 58 | 0 (0–6.2) | 1.0 (1.0–1.0) | |
| 1 month post-primary vaccination | 114 | 82 (73.2–88.2) | 9.55 (8.04–11.0) | |  | 58 | 0 (0–6.2) | 1.02 (0.98–1.06) | |
| Pre-booster vaccination | 114 | 17 (10.3–24.8) | 1.93 (1.62–2.30) | |  | 58 | 2 (0.04–9.2) | 1.05 (0.98–1.12) | |
| 1 month post-booster vaccination | 114 | 94 (87.8–97.5) | 27 (22.0–33.0) | |  | 58 | 0 (0–6.2) | 1.0 (1.0–1.0) | |
| NHBA |  | | |  | | | | |  |
| Baseline (pre-vaccination) | 93 | 12 (6.1–20.2) | 1.45 (1.24–1.69) | |  | 49 | 16 (7.3–29.7) | 1.75 (1.32–2.31) | |
| 1 month post-primary vaccination | 104 | 62 (51.5–70.9) | 8.93 (6.92–12.0) | |  | 54 | 6 (1.2–15.4) | 1.17 (1.01–1.35) | |
| Pre-booster vaccination | 109 | 23 (15.4–32.0) | 2.29 (1.85–2.83) | |  | 52 | 6 (1.2–15.9) | 1.46 (1.21–1.76) | |
| 1 month post-booster vaccination | 110 | 92 (85.0–96.2) | 18 (15.0–22.0) | |  | 58 | 12 (5.0–23.3) | 1.53 (1.27–1.85) | |

hSBA, human serum bactericidal assay; GMT, geometric mean titer; 4CMenB, 4-component serogroup B recombinant meningococcal vaccine; N, number of infants with available results; CI, confidence interval; fHbp, factor H-binding protein; NadA, Neisserial adhesin A; PorA, porin A ; NHBA, *Neisseria* heparin-binding antigen.

# Figure S1. Participants in the 4CMenB+Routine group with hSBA titers ≥4 against each of the indicator strains for the four vaccine antigens (full analysis set)

4CMenB, 4 component serogroup B recombinant meningococcal vaccine; hSBA, human serum bactericidal assay, fHbp, factor H-binding protein; NadA, Neisserial adhesin A; PorA, porin A; NHBA, *Neisseria* heparin-binding antigen.

# Supplementary Text S1

**Exclusion criteria:**

- a history of any meningococcal vaccine administration;
- a prior vaccination with any diphtheria, tetanus, pertussis (acellular or whole cell), polio (either inactivated or oral), *Haemophilus influenzae* type b, pneumococcal, measles, mumps and rubella (MMR) or varicella antigens;
- previously ascertained or suspected disease caused by *Neisseria meningitidis* or a household contact with and/or intimate exposure to an individual with laboratory confirmed *N. meningitidis*;
- a history of severe allergic reaction after previous vaccinations or hypersensitivity to any vaccine component; a significant acute or chronic infection within the previous 7 days or body temperature ≥38°C within the previous day;
- administration of antibiotics within 6 days prior to enrollment;
- any serious chronic or progressive disease according to the judgment of the investigator (eg, neoplasm, insulin dependent diabetes mellitus Type I, cardiac disease, hepatic disease, progressive neurological disease or seizure, either associated with fever or as part of an underlying neurological disorder or syndrome, autoimmune disease, human immunodeficiency virus (HIV) infection or acquired immune deficiency syndrome (AIDS), or blood dyscrasias or diathesis, signs of cardiac or renal failure or severe malnutrition);
- known or suspected impairment/alteration of the immune system, immunosuppressive therapy, use of systemic corticosteroids or chronic use of inhaled high-potency corticosteroids since birth;
- received blood, blood products and/or plasma derivatives or any parenteral immunoglobulin preparation; received or intent to immunize with any other vaccine(s) (with the exception of rotavirus vaccine, influenza vaccine, and second hepatitis B [HepB] vaccine), within 28 days prior and throughout the study period (infants should have received HepB vaccine preferably at 0, 1 month of age, with the second dose at least 14 days prior to study vaccination; influenza vaccine should have been administered at least 14 days before or 14 days after study vaccination; rotavirus vaccine could be administered during the study as per local practice);
- infants who were participating in another clinical trial since birth or plan to participate during the study;
- infants who had family members and household members of research staff; or had any condition which, in the opinion of the investigator, could have interfered with the evaluation of the study objectives.

An infant who met all entry criteria except one related to transient clinical circumstances (body temperature elevation or recent use of excluded medication or vaccine), could have been considered eligible for study enrollment if the appropriate window for delay had passed.
